# Direct health care cost of treatment and medication of biliary atresia patients using the National Database of Health Insurance Claims and Specific Health Checkups

**DOI:** 10.1007/s00383-022-05079-1

**Published:** 2022-02-15

**Authors:** Eri Hoshino, Keiko Konomura, Masayuki Obatake, Kensuke Moriwaki, Michi Sakai, Kevin Y. Urayama, Kojiro Shimozuma

**Affiliations:** 1 Comprehensive Unit for Health Economic Evidence Review and Decision Support (CHEERS), Ritsumeikan University, Kyoto, Japan; 2 Graduate School of Public Health, St. Luke’s International University, Tokyo, Japan; 3 Center for Outcomes Research and Economic Evaluation for Health (C2H), National Institute of Public Health, Saitama, Japan; 4 Department of Pediatric Surgery, Kochi Medical School, Nankoku, Japan; 5 Department of Social Medicine, National Center for Child Health and Development, Tokyo, Japan

**Keywords:** Biliary atresia, Direct health care cost, Health insurance claims in Japan

## Abstract

**Background:**

Treatment of biliary atresia (BA), which typically requires an initial surgical intervention called the Kasai procedure (KP) and possible liver transplant (LT) afterwards, is quite resource-intensive and would affect patients and families for a lifetime; yet a comprehensive view of the economic burden has not been reported. We estimated direct health care costs from the public payer perspective using the National Database of Health Insurance Claims and Specific Health Checkups of Japan.

**Methods:**

Children newly diagnosed at ages 0 days to 4 years between April 2010 and September 2019 were identified. Costs of treatment were estimated for six phases of care: prediagnosis, KP and inpatient hospitalization, follow-up after KP, pre-transplant checkup, LT and inpatient hospitalization, and follow-up after LT.

**Results:**

Mean total prediagnosis medical cost was $6847 (USD) and KP and inpatient hospitalization was $42,157 per year. Follow-up after KP was $15,499, and pre-transplant checkup after KP was $36,015 per year. Mean cost for LT and inpatient hospitalization was $105,334, and follow-up after liver transplant was $25,459 per year.

**Conclusions:**

Treatment of BA requires extensive medical resource consumption. The use of the comprehensive national database allowed us to estimate the costs which will be useful for health service planning and cost-effectiveness analysis.

**Supplementary Information:**

The online version contains supplementary material available at 10.1007/s00383-022-05079-1.

## Introduction

Biliary atresia (BA) is a childhood rare disease of liver and bile ducts that presents with biliary obstruction exclusively in the neonatal period [1]. BA has an estimated incidence of around 0.3–3.7 per 10,000 live births depending on geographic region; the incidence in Japan is higher than most countries with an estimated 1.04–1.1 diagnoses per 10,000 live births [2–6]. BA is the most common cause of neonatal jaundice requiring prompt surgical intervention (the Kasai procedure [KP]) that aims to restore bile flow, and is the most frequent indication for liver transplantation (LT) in children [1]. The therapeutic approach consists of KP as the first strategy and, in case of failure, LT [7]. The timing of surgical intervention with KP is one of the main prognostic factors; therefore, infants with suspected BA should be evaluated as rapidly as possible [8]. Over the past decade, the average age at KP has remained between 60 and 70 days, and the 10-year native liver survival is 52.8% in Japan [9, 10–13]. Even after successful KP, many patients will experience progressive liver disease and the majority of patients with BA will eventually require LT [13, 14]. In Japan, a living donor liver transplant is the most common type of LT due to the lack of deceased donor transplant opportunities. From November 1989 to December 2015, 7862 LTs were performed, of which only 321 cases were from deceased donor liver transplants in Japan [15]. Treatment of BA is resource-intensive and would affect patients and families for their lifetime. Previous research reporting the costs of BA treatment were based on single institutions [16, 17], and a comprehensive perspective of the medical resource use has not been reported.

In Japan, universal health care insurance requires a co-payment of 30% except for elderly people and children [18]. For children, the co-payment is 20% which is eligible to be covered by the Medical Care Certificate for Children issued by the municipalities. The certificate covers direct medical expenses, including treatments and medication until the age of 15. The ceiling limits for age and coverage rate depend on household income levels and differ by regional jurisdictions. Also, the Medical Aid Program for Chronic Pediatric Diseases of Specified Categories allows children under the age of 19 years with chronic diseases, such as BA, opportunities to receive financial assistance based on level of household income [19].

A high standard of medical care, as a result of recent technological advancements, has improved the prognosis of children with chronic diseases leading to longer survival and reduced overall mortality rates. However, this has also been accompanied by higher demands for medical resources for patients over the course of a potentially extended illness. Yet, comprehensive analyses of the medical resource consumption for BA have not been previously reported. Evaluating the uses of medical resources will help to understand the costs related to current mainstream practice and provide a scientific foundation for high-quality cost-effectiveness evaluations. The clinical practice guidelines development manual in Japan recently added a section for health economic evaluation indicating that an economic perspective for clinical practices is important [20]. Several cost-effectiveness studies on population-based BA screening have been published including the calculation of an incremental cost-effectiveness ratio (ICER) [17, 16]. The denominator of the ICER reflects the effect of treatment such as increases in quality-adjusted life-years and the numerator of the ICER is the difference in cost between the treatment options. None of previous cost-effectiveness analyses utilized a national level cost estimate. Especially when estimating a strategy for a population-based approach, costs using aggregated data at a national level is ideal. The objective of this study was to estimate the direct health care costs for the treatment of BA using the National Database of Health Insurance Claims and Specific Health Checkups of Japan (NDB).

## Methods

We conducted a retrospective cohort analysis using the NDB. The cost calculation was based on the public payer point of view. Our primary outcome measure was total direct medical cost of treatment and medication. This study involved the use of de-identified data and was approved by the Institutional Review Board at the National Institute of Public Health (ID#NIPH-IBRA#12306).

### Data

We used the NDB which was launched by the Ministry of Health, Labour and Welfare in April 2009 [21]. The database stores health insurance claims and data for specific health exams performed annually. The NDB contains information such as patient age, gender, diagnosis, inpatient-related medical data, outpatient-related medical data, use of dental services, drug prescriptions, and health checkup data. The NDB comprises datasets of insured medical care which were linked using unique identifiers [22]. We used data from April 2010 to September 2019, including direct medical outpatient, inpatient, and medication costs.

### Patients

We identified BA patient diagnoses based on International Classification of Diseases (ICD)-10 disease name (code: Q44.2) in the NDB for the analysis period of 2010 to 2019, and categorized the patients into two groups; those receiving KP and those receiving LT and diagnosed as LT-related complication. The patients who had both KP and LT were included in the LT group. Potentially erroneous data indicating either of KP or LT had not been performed, and KP at the age of 5 years or older were excluded as we would expect untreated BA to lead to biliary cirrhosis and death within the early years of life. Due to ethical concerns attached to the nature of rare pediatric diseases like BA, the date of birth was not included in the de-identified dataset. Instead, ages were labeled as ordinal categories at 5-year age intervals (0–4 years, 5–9 years, etc.).

For each patient, the following characteristics were available; gender, associated anomaly, the number of KPs performed, length of hospital stay at the time of KP, and length of hospital stay at the time of LT. We categorized associated anomaly into BA with splenic malformation (BASM) including any spleen abnormalities, and non-BASM including cardiac defect and other malformations as previously categorized [23–25]. The list of ICD-10 codes corresponding to each malformation included in our analysis is shown in Supplementary Table 1. Hospital locations where patients received KP or LT were also included, and the locations were categorized into three groups: communities with over 5,000,000 residents (including Tokyo, Kanagawa, Osaka, Aichi, Saitama, Chiba, Hyogo, Hokkaido, Fukuoka), communities with 1,000,000–4,999,999 residents, and communities with less than 1,000,000 residents. Also, the size of hospitals was divided into 3 groups; less than 600 beds, 600–799 beds, more than 800 beds.

### Medical cost

The Japanese health care system requires healthcare institutions to claim medical service fees and receive payments from insurers. The fees charged for medical services and pharmaceuticals are set by the Ministry of Health, Labour and Welfare. The medical costs in this analysis were estimated on a fee-for-service basis [26], and the total amount of fee-for-service was determined by the accumulation of points for individual medical practices and fee schedule. Medical costs were calculated based on the phase care approach which divides long-term treatment into clinically relevant periods, such as the initial treatment period before and after diagnosis, and the intervening or follow-up period [27]. The phases were divided into six stages; prediagnosis, KP and inpatient hospitalization, follow-up after KP, pre-transplant checkup, LT and inpatient hospitalization, and follow-up after LT.

Costs were calculated separately for a total direct medical cost and medication. The total direct medical costs included all of inpatient and outpatient treatment and medication costs. Before diagnosis, the treatment costs included laboratory tests, liver ultrasound, hepatobiliary scintigram, abdominal computed tomography scan, magnetic resonance imaging, endoscopy, duodenal fluid collection, liver biopsy, vibration controlled transient elastography, general outpatient visit fee, inpatient fee, and other related costs. Treatment costs of KP and inpatient hospitalization included emergency department visits, surgery, inpatient hospitalizations, and other related costs. Treatment costs for outpatient follow-up for 1 year after KP included clinical examinations indicated above and general outpatient visit fee. Treatment costs for pre-transplant checkup was calculated based on the medical resource consumption 1 year prior to LT including the more detailed examinations beyond regular check-ups. Treatment costs of LT included the costs for the procedure and items similar to those described for KP. Since almost all LT in Japan are from a living related donor, hospital and outpatient costs for the donor were included in the patients’ costs. Costs of medication included major medications for treating BA such as fat-soluble vitamin supplements, corticosteroids, antibiotics, choleretics, immunosuppressants when LT is indicated, taurine, and inchinkoto (Kampo medicine) and other medications prescribed in inpatient and outpatient settings. The cost of over-the-counter drugs were not included.

Medical service fees set by the Ministry of Health, Labour and Welfare are revised every 2 years to respond to the changing social and economic conditions of the times [28]; prices used in this analysis were adjusted accordingly. The details of the revisions relevant to cost estimations were shown in Supplementary Table 2. Costs were expressed in US dollars (USD, $) after converting from Japanese yen (JPY, ¥) based on the exchange rate as of June 2021 (1 USD = 110 JPY).

### Data analysis

For estimating the incidence of BA in Japan, patients who were newly diagnosed as BA between ages 0 to 4 were summarized by year from 2011 to 2018. We excluded 2010 data to extract patients with the first diagnosis in 2011 to estimate the incidence and 2019 data because it did not have a full year observation (April to September in 2019). The denominator was the number of births in Japan, and the incidence rate was expressed per 10,000 persons using results from the national statistics [29]. Patient characteristics were summarized by proportions of patients within categories between KP and LT. Costs were summarized separately for the total medical cost and medication cost using the mean with SD and 95% confidence interval (95% CI). We used SAS software, version 9.4 for Windows (SAS institute Inc., Cary, NC, USA) for our data analyses.

## Results

### Patient characteristics

**Fig. 1:**
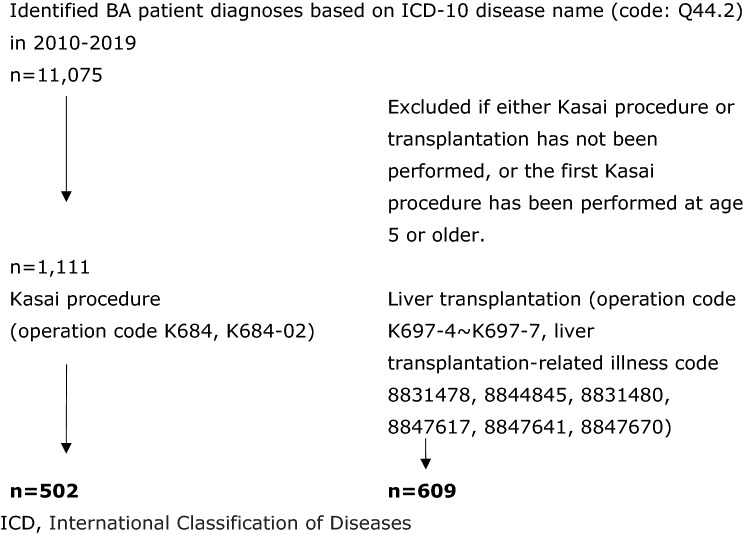
Biliary atresia patients identified from the National Database of Health Insurance Claims and Specific Health Checkups of Japan, 2010–2019. *ICD* International Classification of Diseases

Figure 1 shows the number of BA patients with KP and LT from 2010 to 2019 in the NDB. Overall, 1111 BA patients were identified including a total of 502 patients who had KP and 609 who had LT. Table 1 shows the characteristics of patients. 552 patients underwent KP, among which 298 (59.4%) were girls. A total of 609 patients underwent LT, and 383 patients (62.9%) receiving LT were girls. Both KP and LT were performed in 303 patients. Associated anomaly was observed in 80 patients who underwent KP, and 10 patients (2.0%) were recognized as BASM. More than half of KPs were performed in hospitals located in areas with over 5,000,000 residents (Tokyo, Kanagawa, Osaka, Aichi, Saitama, Chiba, Hyogo, Hokkaido, Fukuoka), and 125 (24.9%) were performed at large capacity hospitals (> 800 beds). The mean length of hospital stay was 65.4 days (SD ± 44.4) for those who only had KP performed. For those who received KP and LT, the mean length of hospital stay was 113.2 days (SD ± 118.5) for KP and 200.7 days (SD ± 124.4) for LT.

**Table 1:** Characteristics of biliary atresia patients identified from the National Database of Health Insurance Claims and Specific Health Checkups of Japan, 2010–2019

Variables	Kasai procedure, *n* = 502		Liver transplantation, *n* = 609	
	*n* (%)		*n* (%)	
Age group at first diagnosis (years)				
0–4	502 (100)		470 (77.2)	
5–9	–		34 (5.6)	
10–14	–		49 (8.0)	
15–19	–		56 (9.2)	
Male	204 (40.6)		226 (37.1)	
Female	298 (59.4)		383 (62.9)	
Associated anomaly				
Non-BASM	80 (15.9)		117 (19.2)	
BASM	10 (2.0)		12 (2.0)	
Number of KP performed				
0	–		306 (50.2)	
> 1	502 (100)		303 (49.8)	
Hospital locations, Number of residents				
> 5,000,000	279 (55.6)		156 (25.6)	
1,000,000–4,999,999	185 (36.9)		125 (20.5)	
< 1,000,000	38 (7.6)		22 (3.6)	
Hospital size, Number of beds				
< 600	90 (17.9)		83 (13.6)	
600–799	53 (10.6)		43 (7.1)	
> 800	125 (24.9)		78 (12.8)	

	*n*	Mean (SD)	*n*	Mean (SD)
Days at hospital at KP	482	65.4 (44.4)	293	113.2 (118.5)
Days at hospital at LT	–	–	21	200.7 (124.4)

*BASM* biliary atresia with splenic malformation, *KP* Kasai procedure, *LT* liver transplantation, *SD* standard deviation;

*For the liver transplantation, the number represents those who had Kasai procedures one or more times

### Incidence

Table 2 shows the number of newly diagnosed BA in each year from 2011 to 2018. The lowest incidence was observed in 2016 at 1.05 per 10,000 births, and the highest was observed in 2018 at 1.47 per 10,000 births.

**Table 2:** Crude Incidence of biliary atresia from 2011 to 2018 in Japan

Year	Number of patients	Number of births	Incidence */*10,000
2011	130	1,050,807	1.24
2012	122	1,037,232	1.18
2013	111	1,029,817	1.08
2014	119	1,003,609	1.19
2015	125	1,005,721	1.24
2016	103	977,242	1.05
2017	111	946,146	1.17
2018	135	918,400	1.47

### Medical costs

Table 3 shows the annual direct medical costs and medication expressed according to each phase of care. The mean total prediagnosis medical cost including inpatient and outpatient expenditure was $6847 (USD). The cost for KP and inpatient hospitalization was $42,157 per year. The follow-up after KP for inpatient and outpatient cost was $15,499, and pre-transplant checkup after KP was $36,015 per year. The mean cost for LT and inpatient hospitalization was $105,334, and follow-up after liver transplant including impatient and outpatient was $25,459 per year.

**Table 3:** Annual direct medical cost of treatment and medication for biliary atresia in 2021 US dollars

	(a) Total treatment cost			(b) Medication cost			(a)/(b)
	*n*	Mean (SD)	95% CI	*n*	Mean (SD)	95% CI	%
Kasai procedure (age 0–4)							
Prediagnosis, outpatient	163	403 (1996)	94–711	163	83 (415)	19 to 148	20.7
Prediagnosis, inpatient	178	6444 (9449)	5046–7842	178	143 (386)	86 to 200	2.2
KP and inpatient hospitalization	443	42,157 (20,216)	40,269–44,044	443	1544 (2031)	1354 to 1733	3.7
Follow-up after KP, outpatient	428	1781 (2487)	1544–2017	428	902 (2186)	694 to 1110	50.7
Follow-up after KP, inpatient	293	13,718 (15,190)	11,971–15,464	293	672 (1004)	557 to 788	4.9
Liver transplantation (age 0–19)							
Pre-transplant checkup, outpatient	21	2080 (4166)	192–3,985	21	766 (1664)	8 to 1523	36.7
Pre-transplant checkup, inpatient	20	33,935 (30,148)	19,826–48,045	20	1741 (3043)	317 to 3166	5.1
LT and inpatient hospitalization	24	105,334 (34,750)	90,660–120,007	24	11,967 (10,268)	7631 to 16,302	11.4
Follow-up after LT, outpatient	22	8051 (5135)	5774–10,327	22	5889 (4625)	3838 to 7940	73.2
Follow-up after LT, inpatient	17	17,408 (25,441)	4328–30,489	17	4963 (15,158)	− 2831 to 12,757	28.5

*SD* standard deviation, *CI* confidence interval, *IQR* interquartile range, *KP* Kasai procedure, *LT* liver transplantation

**Fig. 2:**
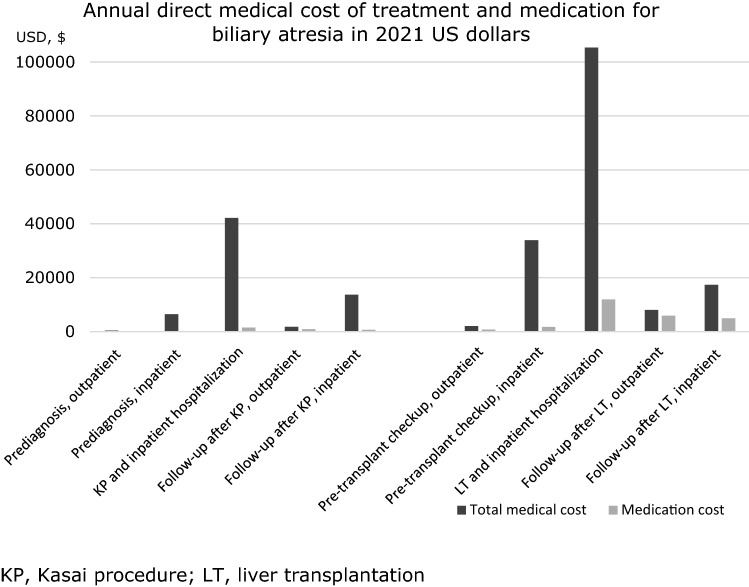
Mean annual direct medical cost of treatment and medication, in 2021 US dollars. *KP* Kasai procedure, *LT* liver transplantation

The most cost-intensive care phase was when patients received LT and hospitalization (total mean medical cost = $105,334), followed by KP and hospitalization (mean = $42,157). In general, medication cost accounts for a higher percentage of the total treatment cost in outpatient setting (20.7%-73.2%) than in inpatient setting (2.2%-28.5%). Figure 2 shows that in general, the cost of medication increased dramatically after LT. The most cost-intensive phase for medication spending was at LT and hospitalization (mean = $11,967), followed by outpatient follow-up after LT (mean = $5889).

## Discussion

This is the first comprehensive analysis of medical resource consumption related to the treatment of BA in Japan, in which we had the opportunity to access high-quality data through the NDB. In our analysis, data for a large number of patients were available for the examination of KP-related costs, but fewer numbers were available for LT. LT registration procedures are already mandatory under the Japanese Liver Transplantation Society and are validated by the national registry of the Japanese Transplantation Society and the national clinical database of the Japan Surgical Society [30]. According to the Japanese Liver Transplantation Society, the annual number of pediatric LT cases ranged from 115 to 145 between 2009 and 2015 [15].

In our study, a total of 609 patients underwent LT from April 2010 to September 2019 which is equivalent to about 60–65 patients annually. However, the cost data were available for only 24 LT patients. The possible explanation for this low number representing LT patients in the NDB may be attributed to the practice of many hospitals managing LT cost issues at the institutional level and non-electrically. In Japan, a medical institution that receive organs from the deceased for transplant must be fully registered as a member of the Japan Organ Transplant Network (JOT) [31]. As of April 2000, 25 facilities were registered as accredited liver transplant facilities and have the capability to also perform living donor liver transplants. Registration requires the facility to have sufficient experience in living donor liver transplantation and have a high-quality operation system in place. When LT is performed at a JOT-registered institution, it requests reimbursement for LT-related management fees, and the payment is made to the donor facility via JOT according to the cost allocation rules defined by JOT. Due to this additional billing and LT management system, it is possibility that not all cases are reflected in the NDB. For the purpose of estimating medical cost of BA, we cannot rule out the potential influences of the underrepresented cases.

Our study showed the incidence of BA in Japan to be about 1.05 to 1.47 per 10,000 live births while previous studies have reported incidence in various countries to range between 0.3 and 3.7 per 10,000 live births with estimates for Japan being about 1.04–1.10 per 10,000 [2]. The NDB is considered to have sufficiently captured a patient population that represents the national patient cohort.

The treatment costs incurred over a lifetime differ significantly depending on whether or not LT is performed. Furthermore, the inclusion of pre- and post-liver transplant examination and management costs, in addition to the medical costs of LT itself, would affect the total cost dramatically. In a cost-effectiveness analysis, it is expected that the total life-time medical costs will differ depending on the age at the time of LT. This is consistent with previous reports that the age at transplantation is a predictor for the total cost of treatment [32]. Our findings also suggests that the total cost depends on the disease state which requires different care at different ages.

In general, the average number of days spent in hospitals by patients in Japan is greater than in the U.S. and Europe [33]. Our study supports this showing that the average length of hospitalization after KP is 65.4 days, considerably longer than any other country which ranges from about 10 to 20 days [34]. This contributes to an enormous increase in national medical care expenditures as costs for inpatient treatments are greater than those for outpatient treatments. However, since the medical service fee is set and strictly controlled by the government, the unit cost for inpatient treatment may be lower in Japan compared to Western countries resulting in relatively compatible total costs of treatment. Studies based on a single institution in Canada and the U.S. showed estimates of 23,466 Canadian dollars (CAD; equivalent to 18,746 USD) for KP and 452,635 CAD (equivalent to 361,594 USD) for LT, and 61,248 USD for treatment without LT and 187,382 USD for LT, respectively, [17, 16]. For Japan, our results showed costs of 42,157 USD for KP and its hospitalization which was higher than Canada but lower than the U.S., and 105,334 USD for LT and its hospitalization which was lower than both Canada and the U.S.

We acknowledge the limited representation of patients undergoing LT used in our cost estimations. Although we were not able to capture an entirely population-based series of LT, results derived from the subset included in the NDB provided a valid estimate of cost experienced by these series of patients who were processed through the national insurance system. Also, a limitation of our study is that the costs estimation is based only on data from the publicly financed health care system, and direct non-medical cost, out-of-pocket cost, and opportunity costs for patients and caregivers are not included. Our costs are based on the medical practice in Japan which may differ across different regions of the world. Medical practice within Japan may have changed over the years and we estimated resource use and costs averaged over the entire study period. In 2018, the clinical guideline for the treatment of BA was published in Japan [35] leading to more standardized clinical practice that may influence changes in medical expense in the future. Even though we used the phase of care approach to estimate direct medical costs according to clinically relevant periods, the disease severity within each phase was unknown.

### Conclusion

Treatment of BA requires extensive medical resource consumption, and costs vary greatly depending on the phase of treatment. The use of the comprehensive national database allowed us to estimate the costs, the results of which may be useful for future health services planning and cost-effectiveness analysis.

## Supplementary Information

Below is the link to the electronic supplementary material.

Supplementary file1 (DOCX 51 KB)
