# Supplementary material for: Direct health care cost of treatment and medication of biliary atresia patients using the National Database of Health Insurance Claims and Specific Health Checkups
Source: Pediatr Surg Int. 2022 Feb 15;38(4):547–54. doi: 10.1007/s00383-022-05079-1 (PMC8913443; doi:10.1007/s00383-022-05079-1)
Supplement: Supplementary file 1 — Supplementary file1 (DOCX 51 KB) [file 383_2022_5079_MOESM1_ESM.docx]

| Identified BA patient diagnoses based on ICD-10 disease name (code: Q44.2) in 2010-2019 | | |
| --- | --- | --- |
| n=11,075 |  |  |
|  |  | Excluded if either Kasai procedure or transplantation has not been performed, or the first Kasai procedure has been performed at age 5 or older. |
| n=1,111 |  |  |
| Kasai procedure  (operation code K684, K684-02) |  | Liver transplantation (operation code K697-4~K697-7, liver transplantation-related illness code 8831478, 8844845, 8831480, 8847617, 8847641, 8847670) |
| **n=502** |  | **n=609** |

ICD, International Classification of Diseases

Figure 1 Biliary atresia patients identified from the National Database of Health Insurance Claims and Specific Health Checkups of Japan, 2010-2019

Table 1 Characteristics of biliary atresia patients identified from the National Database of Health Insurance Claims and Specific Health Checkups of Japan, 2010-2019

| **Variables** |  | **Kasai procedure,**  **n=502** | | **Liver transplantation,**  **n=609** | |
| --- | --- | --- | --- | --- | --- |
|  |  | n (%) | | n (%) | |
| **Age group at first diagnosis (years)** | 0-4 | 502 (100) | | 470 (77.2) | |
|  | 5-9 | - | | 34 (5.6) | |
|  | 10-14 | - | | 49 (8.0) | |
|  | 15-19 | - | | 56 (9.2) | |
|  | | | | | |
| **Male** |  | 204 (40.6) | | 226 (37.1) | |
| **Female** |  | 298 (59.4) | | 383 (62.9) | |
|  | | | | | |
| **Associated anomaly** | non-BASM | 80 (15.9) | | 117 (19.2) | |
|  | BASM | 10 (2.0) | | 12 (2.0) | |
|  | | | | | |
| **Number of KP performed** | 0 | - | | 306 (50.2) | |
|  | >1 | 502 (100) | | 303 (49.8) | |
|  | | | | | |
| **Hospital locations, Number of residents** | > 5,000,000 | 279 (55.6) | | 156 (25.6) | |
|  | 1,000,000 -4,999,999 | 185 (36.9) | | 125 (20.5) | |
|  | <1,000,000 | 38 (7.6) | | 22 (3.6) | |
|  | | | | | |
| **Hospital size, Number of beds** | <600 | 90 (17.9) | | 83 (13.6) | |
|  | 600-799 | 53 (10.6) | | 43 (7.1) | |
|  | >800 | 125 (24.9) | | 78 (12.8) | |
|  | | | | | |
|  |  | n | Mean (SD) | n | Mean (SD) |
| **Days at hospital at KP** |  | 482 | 65.4 (44.4) | 293 | 113.2 (118.5) |
| **Days at hospital at LT** |  | - | - | 21 | 200.7  (124.4) |

BASM, biliary atresia with splenic malformation; KP, Kasai procedure; LT, liver transplantation SD, standard deviation;

* For the liver transplantation, the number represents those who had Kasai procedures one or more times.

Table 2 Crude Incidence of biliary atresia from 2011 to 2018 in Japan

| **Year** | **Number of patients** | **Number of births** | **Incidence** */***10,000** |
| --- | --- | --- | --- |
| **2011** | 130 | 1,050,807 | 1.24 |
| **2012** | 122 | 1,037,232 | 1.18 |
| **2013** | 111 | 1,029,817 | 1.08 |
| **2014** | 119 | 1,003,609 | 1.19 |
| **2015** | 125 | 1,005,721 | 1.24 |
| **2016** | 103 | 977,242 | 1.05 |
| **2017** | 111 | 946,146 | 1.17 |
| **2018** | 135 | 918,400 | 1.47 |

Table 3 Annual direct medical cost of treatment and medication for biliary atresia in 2021 US dollars

|  | 1. **Total treatment cost** | | |  | 1. **Medication cost** | | | **(a)/(b)** |
| --- | --- | --- | --- | --- | --- | --- | --- | --- |
|  | **n** | **Mean**  **(SD)** | **95% CI** |  | **n** | **Mean**  **(SD)** | **95% CI** | **%** |
| **Kasai procedure (age 0-4)** | | | |  |  | | |  |
| **Prediagnosis, outpatient** | 163 | 403 (1,996) | 94-711 |  | 163 | 83 (415) | 19-148 | 20.7 |
| **Prediagnosis, inpatient** | 178 | 6,444(9,449) | 5,046-7,842 |  | 178 | 143 (386) | 86-200 | 2.2 |
| **KP and inpatient hospitalization** | 443 | 42,157 (20,216) | 40,269-44,044 |  | 443 | 1,544 (2,031) | 1,354-  1,733 | 3.7 |
| **Follow-up after KP, outpatient** | 428 | 1,781(2,487) | 1,544-2,017 |  | 428 | 902 (2,186) | 694-1,110 | 50.7 |
| **Follow-up after KP, inpatient** | 293 | 13,718 (15,190) | 11,971-15,464 |  | 293 | 672  (1,004) | 557-788 | 4.9 |
| **Liver transplantation (age 0-19)** | | | |  |  | | |  |
| **Pre-transplant checkup, outpatient** | 21 | 2,080(4,166) | 192-3,985 |  | 21 | 766 (1,664) | 8-1,523 | 36.7 |
| **Pre-transplant checkup, inpatient** | 20 | 33,935 (30,148) | 19,826-48,045 |  | 20 | 1,741 (3,043) | 317-3,166 | 5.1 |
| **LT and inpatient hospitalization** | 24 | 105,334 (34,750) | 90,660-120,007 |  | 24 | 11,967 (10,268) | 7,631-16,302 | 11.4 |
| **Follow-up after LT, outpatient** | 22 | 8,051 (5,135) | 5,774-10,327 |  | 22 | 5,889 (4,625) | 3,838-7,940 | 73.2 |
| **Follow-up after LT, inpatient** | 17 | 17,408 (25,441) | 4,328-30,489 |  | 17 | 4,963 (15,158) | -2,831-12,757 | 28.5 |

SD, standard deviation; CI, confidence interval; IQR, interquartile range; KP, Kasai procedure; LT, liver transplantation

USD, $

KP, Kasai procedure; LT, liver transplantation

Figure 2 Mean annual direct medical cost of treatment and medication, in 2021 US dollars

Supplementary Table 1. List of ICD-10 codes for associated anomaly

| ICD-10 code | Description |
| --- | --- |
| Q210 | Ventricular septal defect |
| Q211 | Atrial septal defect |
| Q212 | Atrioventricular septal defect |
| Q213 | Tetralogy of Fallot |
| Q250 | Patent ductus arteriosus |
| Q256 | Stenosis of pulmonary artery |
| Q262 | Total anomalous pulmonary venous connection |
| Q268 | Other congenital malformations of great veins |
| Q410 | Congenital absence, atresia and stenosis of duodenum |
| Q423 | Congenital absence, atresia and stenosis of anus without fistula |
| Q433 | Congenital malformations of intestinal fixation |
| Q434 | Duplication of intestine |
| Q890 | Congenital absence and malformations of spleen |

Supplementary Table 2. Revision of medical fee service and drug prices, % change

| Year | % change |
| --- | --- |
| 2008→2010 | 1.0019 |
| 2010→2012 | 1.00004 |
| 2012→2014 | 1.001 |
| 2014→2016 | 0.9916 |
| 2016→2018 | 0.9881 |
| 2018→2020 | 0.9954 |
